# Biologically active compounds from marine organisms in the strategies for combating coronaviruses

**DOI:** 10.3934/microbiol.2020028

**Published:** 2020-12-07

**Authors:** Tatyana S. Zaporozhets, Nataliya N. Besednova

**Affiliations:** Immunology Laboratory, Somov Institute of Epidemiology and Microbiology, Vladivostok, Russian Federation

**Keywords:** coronaviruses, marine organisms, biologically active substances, antiviral drugs, SARS-CoV, MERS-CoV, SARS-CoV-2

## Abstract

Despite the progress made in immunization and drug development, so far there are no prophylactic vaccines and effective therapies for many viral infections, including infections caused by coronaviruses. In this regard, the search for new antiviral substances continues to be relevant, and the enormous potential of marine resources are a stimulus for the study of marine compounds with antiviral activity in experiments and clinical trials. The highly pathogenic human coronaviruses-severe acute respiratory syndrome-related coronavirus (SARS-CoV), Middle East respiratory syndrome coronavirus (MERS-CoV), severe acute respiratory syndrome-related coronavirus 2 (SARS-CoV-2) remain a serious threat to human health. In this review, the authors hope to bring the attention of researchers to the use of biologically active substances of marine origin as potential broad-spectrum antiviral agents targeting common cellular pathways and various stages of the life cycle of different viruses, including coronaviruses. The review has been compiled using references from major databases such as Web of Science, PubMed, Scopus, Elsevier, Springer and Google Scholar (up to June 2020) and keywords such as ‘coronaviruses’, ‘marine organisms’, ‘biologically active substances’, ‘antiviral drugs’, ‘SARS-CoV’, ‘MERS-CoV’, ‘SARS-CoV-2’, ‘3CLpro’, ‘TMPRSS2’, ‘ACE2’. After obtaining all reports from the databases, the papers were carefully analysed in order to find data related to the topic of this review (98 references). Biologically active substances of marine origin, such as flavonoids, phlorotannins, alkaloids, terpenoids, peptides, lectins, polysaccharides, lipids and others substances, can affect coronaviruses at the stages of penetration and entry of the viral particle into the cell, replication of the viral nucleic acid and release of the virion from the cell; they also can act on the host's cellular targets. These natural compounds could be a vital resource in the fight against coronaviruses.

## 1. Introduction

In the modern era, among the many infectious threats that people face, viruses pose a serious danger, threatening devastating global pandemics. The rapidly changing global landscapes, local environments, the huge population growth, and urbanization in many developing countries and progress in the development of transport have created greater opportunities for the emergence and spread of viral diseases.

At the end of 2019, an ongoing outbreak of pneumonia was first identified in Wuhan, China caused by the novel virus, previously called the 2019-novel coronavirus (2019-nCoV). The International Committee on Taxonomy of Viruses formally associated this virus with severe acute respiratory syndrome coronaviruses (SARS-CoVs), thus designating it as severe acute respiratory syndrome coronavirus 2 (SARS-CoV-2) [1]. On 11 February 2020, the World Health Organization announced that the respiratory disease caused by 2019-nCoV had been officially named Coronavirus Disease 2019 (COVI-19), and on 11 March 2020 described the worldwide spread of the disease as a pandemic [2].

The unprecedented events of the pandemic have exacerbated the overall challenges of controlling viral infections. Despite the progress made in immunization and drug development, so far there are no prophylactic vaccines and effective antiviral therapies for many viral infections, including coronavirus infections [3]. In this regard, the search for new antiviral substances continues to be relevant.

Naturally occurring substances continue to be one of the main sources for prototypes of antimicrobial and antiviral drugs [4]. Over a thousand of novel marine compounds isolated from marine organisms are being pharmacologically tested, and over forty are being existed in the medicine market [5]. Several chemical classes of natural biologically active substances, such as flavonoids, alkaloids and peptides, have been successfully tested against COVID-19 [6].

The world experience of marine pharmacy testifies to the huge potential of marine organisms as raw materials for creating original pharmaceutical substances and drugs [7],[8]. Over the course of evolution, marine organisms have developed many anti-infective strategies and molecules that protect them from the attacks of microbes and viruses that inhabit the marine environment [9]. Compounds isolated from marine organisms capable of inhibiting DNA and RNA viruses, including coronaviruses, have been found among various structural classes such as polysaccharides, terpenoids, steroids, alkaloids, peptides, etc. [9]–[14]. The diversity of these chemical classes is related to the different mechanism used by each of them to inhibit coronaviruses.

**Table 1.** Anti-CoV effects of biologically active compounds from marine organisms and their possible mechanisms.

Source	Compound	Reported antiviral mechanism	Chemical class	First author (yr)
Marine sponge *Aplysinidae*	Fistularin-3/11-epi-fistularin-3 C_31_H_30_Br_6_N_4_O_11_ (PubChem CID 11170714)	Binding with SARS-COV-2^Mpro^, E_score2 = –7,8	Alkaloid	Khan (2020) [5] Felix (2017) [59]
Marine sponge *Aplysinidae*	15-methyl-9(Z)-hexadecenoic acid C_19_H_40_O_3_ (PubChem CID 21646261)	Binding with SARS-COV-2^Mpro^, E_score2 = –7,5	Lipid	Khan (2020) [5] Felix (2017) [59]
Soft coral *Pterogorgia citrina*	(Hexadecyloxy) propane,1,2-diol C_16_H_30_O_2_ (PubChem CID 45638)	Binding with SARS-COV-2^Mpro^, E_score2 = –7,54	Lipid	Khan (2020) [5] Felix (2017) [59]
Brown algae *Sargassum spinuligerum*	Heptafuhalol A	Binding with SARS-COV-2^Mpro^, Δ*G*_B_ = −15,4 kcal/mol	Phlorotannin	Gentile (2020) [12]
	Phlorethopentafuhalol A	Binding with SARS-COV-2^Mpro^, Δ*G*_B_ = −14 kcal/mol	Phlorotannin	
	Pseudopentafuhalol B	Binding with SARS-COV-2^Mpro^, Δ*G*_B_ = −14,6 kcal/mol	Phlorotannin	
	Pseudopentafuhalol C	Binding with SARS-COV-2^Mpro^, Δ*G*_B_ = −14,5 kcal/mol	Phlorotannin	
	Hydroxypentafuhalol A	Binding with SARS-COV-2^Mpro^, Δ*G*_B_ = −14,6 kcal/mol	Phlorotannin	
Marine sponge *Theonella swinhoei*	Pseudotheonamid A	Binding with SARS-COV-2^Mpro^, Δ*G*_B_ = −14,0 kcal/mol	Peptide	Gentile (2020) [12]
	Pseudotheonamid C	Binding with SARS-COV-2^Mpro^, ΔG_B_ = −14,5 kcal/mol	Peptide	
Marine sponge *Petrosia strongylophora sp*.	15-α-methoxypuupehenol C_21_H_26_O_3_ (PubChem CID 460087)	Binding with SARS-COV-2^Mpro^, E score = –7,26	Phenol	Khan (2020) [5] Felix (2017) [59]
Marine sponge *Petrosia strongylophora* sp.	Puupehedione C_22_H_32_O_4_ (PubChem CID 21591485)	Binding with SARS-COV-2^Mpro^, E score = –7,26	Terpene	Khan (2020)[5] Felix (2017) [59]
Brown algae *Sargassum spinuligerum*	Apigenin-7-O-neohesperidoside	Binding with SARS-COV-2^Mpro^, ΔG_B_ = −12,4 kcal/mol	Flavonoid	Gentile (2020) [12]
	Luteolin-7-rutinoside	Binding with SARS-COV-2^Mpro^, ΔG_B_ = −12,1 kcal/mol	Flavonoid	
	Resinoside	Binding with SARS-COV-2^Mpro^, ΔG_B_ = −12,2 kcal/mol	Flavonoid	
Brown algae *Ecklonia cava*	Dieckol (6,6′-bieckol)	Binding with SARS- COV-2^Mpro^, ΔG_B_ = −12,0 kcal/mol	Phlorotannin	Gentile (2020) [12]
Red marine algae *Griffithsia* sp.	Griffithsin	Inhibits viral replication and the cytopathicity induced by SARS, EC_50_ = 0,28 µM	Lectin	Ziółkowska(2006) [13]
Red marine algae *Griffithsia* sp.	Griffithsin	Binds with SARS-CoV (Urbani strain), EC_50_ = 0.61 µg/mL	Lectin	O'Keefe (2010) [22]
		Binds with HCoV-NL63 spike glycoproteins, EC_50_ < 0.0032 µg/mL		
		Binds with HCoV-229E carbohydrates, EC_50_ = 0.16 µg/ mL		
		Binds with HCoV OC43 carbohydrates, EC_50_ = 0.16 µg/mL		
Brown algae *Saccharina japonica*	Sulphated polysaccharide RPI-27	Binds to the S-protein SARS-CoV-2, EC_50_ = 8.3µg/mL	Polysaccharide	Kwon (2020) [26]
	Sulphated polysaccharide RPI-28	Binds to the S-protein SARS-CoV-2, EC_50_ = 1.2 µM	Polysaccharide	
Red marine algae	Iota-carrageenan	Inhibis SARS-CoV-2 replication, IC_50_ = 2.58 µg/mL	Polysaccharide	Morokutti-Kurz (2020) [27]
		Inhibis HCoV OC43 replication, IC_5_ = 0.33 µg/mL		
	Kappa-carrageenan	Inhibis SARS-CoV-2 replication, IC_50_ > 10 µg/mL	Polysaccharide	
		Inhibis hCoV OC43 replication, IC_50_ > 100 µg/mL		
Green algae *Dictyosphaeria versluyii*	Decalactone 4-dictyosphaeric acid A	Binds with TMPRSS2, ΔG_B_ = −14.02	Coumarin	Rahman (2020) [45]
Soft coral *Formosan gorgonian Briareum*	Excavatolide	Binds with TMPRSS2, ΔG_B_ = −14.38	Terpene	Rahman (2020) [45]
Brown algae *Ecklonia cava*	Dieckol	Inhibits SARS-COV-2^3CLpro^, IC_50_ = 68.1 µM	Phlorotannin	Park (2013) [58]
	Dioxinodehydroeckol	Inhibits SARS-COV-2^3CLpro^, IC_50_ = 146.5 µM	Phlorotannin	
	2-phloroeckol	Inhibits SARS-COV-2^3CLpro^, IC_50_ = 112.2 µM	Phlorotannin	
	7-phloroeckol	Inhibits SARS-COV-2^3CLpro^, IC_50_ = 112.0 µM	Phlorotannin	
	Fucodiphloroethol G	Inhibits SARS-COV-2^3CLpro^, IC_50_ = 177.1 µM	Phlorotannin	
Marine sponge *Axinella cf. corrugata*	Esculetin-4-carboxylic acid ethyl ester (C_24_H_20_O_12_Na)	Inhibits SARS-COV-2^3CLpro^, ID_50_ = 46 mmol/L	Coumarin	De Lira (2007) [63]
Marine cyanobacteria	Gallinamide A	Inhibits cathepsin L, IC_50_ = 5.0 nM	Peptide	Miller (2014) [74]
Marine cyanobacteria	Grassistatin A	Inhibits cathepsins D, IC_50_ = 26.5 nM	Peptide	Kwan (2009) [75]
		Inhibits cathepsins E, IC_50_ = 886 pM		
	Grassistatin B	Inhibits cathepsins D, IC_50_ = 7.27 nM	Peptide	
		Inhibits cathepsins E, IC_50_ = 354 pM		
	Grassistatin C	Inhibits cathepsins D, IC_50_ = 1.62 µM	Peptide	
		Inhibits cathepsins E, IC_50_ = 42.9 nM		
Marine sponge *Theonella swinhoei*	Miraziridine A	Inhibits cathepsin L, 60% inhibition at 100 µg/m L,	Peptide	Tabares (2012) [77]
Marine sponge *Theonella aff. mirabilis*	Tokaramide A	Inhibits cathepsin B, IC_50_= 29.0 ng/mL	Peptide	Fusetani (1999) [78]
Marine sponge *Plakortis halichondroides*	Plakortide E C_21_H_34_O	Inhibits SARS PL^pro^, 68% inhibition at 100 µg/mL	Dioxolan	Oli (2014) [79]
		Inhibits cathepsins B, 90% inhibition at 100 µg/mL		
		Inhibits cathepsins L, 85% inhibition at 100 µg/mL		
		Inhibits SARS^Mpro^, 30% inhibition at 100 µg/mL		
Axinellae *polypoides* cultivated from *Streptomyces axinellae*	Tetromycin B	Inhibits cathepsin L, IC_50_ = 32.50 µM	Tetronic acid-based Antibiotic	Pimentel-Elardo (2011) [81]

Note: Mpro: 3CLpro- SARS-CoV-2 Main Protease (3CLpro/Mpro); PLpro: coronaviralpapain-like protease; TMPRSS2: transmembrane serine protease type 2; Δ*G*_B_: the binding free energy; E score: the scoring function, obtained with docking; EC_50_: half maximal effective concentration; IC_50_: 50% inhibitory concentration.

This review provides information on the possibility of using of biologically active compounds from marine organisms of various chemical classes for infections caused by coronaviruses and acting at all stages of the life cycle of viruses. Bacteria, algae, invertebrates (sponges, ophiuras, echinoderms, molluscs, soft corals, bryozoans, and tunnels) and other organisms are sources of new pharmacological compounds of marine origin. The results of the study of the anti-CoV effects of biologically active compounds from marine organisms and the possible mechanisms of their action are presented in Table 1.

## 2. Coronaviruses

Coronaviruses (lat. *Coronaviridae*) are a family of RNA-containing viruses that are combined into two subfamilies: *Coronavirinae* and *Torovirinae* [15]. Within the subfamily *Coronavirinae* are four genera, the alpha, beta, gamma, and delta coronaviruses. Human coronaviruses include HCoV-229E, HCoV-NL63, HCoV-OC43, HCoV-HKU1, SARS-CoV, MERS-CoV, and SARS-CoV-2 [16]. The coronavirus genome is packaged inside a spiral capsid composed of genomic RNA associated with a nucleoprotein (N) surrounded by an envelope. Three structural proteins are associated with the viral envelope: the membrane protein (M) and the envelope protein (E) are involved in virus assembly and the spike protein (S) mediates the entry of the virus into host cells. The coronavirus spike contains three segments: a large ectodomain, a transmembrane anchor, and a short intracellular tail. The ectodomain consists of the receptor-binding subunit S1 and the membrane-fusion subunit S2 [15].

## 3. Life cycle of coronaviruses and targets for the development of antiviral agents

Coronavirus multiplication occurs in several stages. The first stage, the infection phase, includes events leading to adsorption, the penetration of the virus into the cell, the release of the viral genome (deproteinization) and its modification in such a way that it becomes capable of causing the development of infection. The second stage includes transcription, translation, replication of the genome, assembly of the virion components and the exit of the virus from the cell [16].

The initial attachment of the virion to the host cell is initiated by interactions between the S protein and its receptor. The S protein–receptor interaction is the primary determinant for infection of a host species with a coronavirus and also governs the tissue tropism of the virus. S1 ensures the attachment of the virus, and S2 participates in the fusion of the membranes of the host and the virus [16].

Following receptor binding, the virus must gain access to the cytosol of the host cell. This is generally accomplished by acid-dependent proteolytic cleavage of the S protein by cathepsins, transmembrane serine protease type 2 (TMPRSS2) or another protease, followed by fusion of the viral and cellular membranes [16]. The S protein also mediates fusion between lipids of the viral envelope and the plasma membrane of the host cell or membranes of endocytic vesicles to promote delivery of viral genomic RNA into the cytoplasm [16].

The next step is the replication of genomic RNA with the formation of subgenomic RNA used for the synthesis of various proteins that make up the elements of new viral particles: the spike S-protein, envelope E-protein, membrane M-protein and N-nucleocapsid protein. The proteins of the spike, envelope and membrane enter the endoplasmic reticulum, and the nucleocapsid protein combines with the strand of genomic positive RNA, turning into a nucleoprotein complex. They fuse into a complete viral particle in the Golgi apparatus and are excreted into the extracellular region through the vesicle [16].

The life cycle of a virus presents many potential targets for antiviral intervention. Approaches to the development of anti-coronavirus drugs include exposure to the virus during the steps of penetration and entry of a viral particle into a cell, replication of viral nucleic acid, release of virion from a cell, and effects on the cellular targets of the host.

### 3.1. Virus entry into the host cell

Receptor binding and membrane fusion are the initial and critical steps in the coronavirus infection cycle and serve as the main goals in drug development [16]. Penetration usually begins with relatively nonspecific interactions between the virus and attachment factors on the cell surface, followed by the involvement of more specific cellular receptors. Unspecific interaction increases the local concentration of viral particles leading to more effective infection rates. Attachment and penetration inhibitors can act through several inhibitory mechanisms, including binding to virus receptor molecules on the surface of sensitive cells, binding to specific proteins directly in the virion, and also binding to an intermediate, ‘activated’ form of viral protein, preventing further conformational changes [17]. Attachment and penetration inhibitors can serve as a rational basis for antiviral drugs, especially those that can be used in preventive situations. Such compounds reduce the possibility of virus replication at the very first stage and can potentially be less toxic, since the ability to penetrate the membrane may not be required. Although, in practice, such properties are most likely necessary for effective access of the drug through the mucous membranes [18]. However, the main advantage of using penetration inhibitors for emerging viruses is the prevention of the high level of their content in the host cell, which is required by many of these pathogens in order for infection to occur [14].

#### 3.1.1. Inhibitors of the unspecific interaction of the virus to attachment factors on the cell surface

##### 3.1.1.1. Lectins

Lectins, non-immunoglobulin-type proteins capable of recognizing and reversibly binding to carbohydrate fragments of complex glycoconjugates, are potential inhibitors of the attachment of viruses to the surface of sensitive cells without altering their covalent structure [7]. Unlike antiviral drugs, which are based on suppression of the life cycle of the virus, lectins are aimed at preventing penetration of the virus into host cells and further spread of the virus [8]. This group of molecules can affect intercellular interactions and inhibit both cell adhesion and intracellular glycoprotein translocation [19]. The molecular interactions between lectin and its carbohydrate substrate can be highly specific, recognizing both monomeric sugars as well as oligosaccharides formed as part of branched high mannose or complex glycans [19].

Mannose-binding lectins (belonging to the C-type pattern recognition lectins) are a priority among all lectins for research focused on the search for antiviral compounds because of their ability to interrupt self-assembly of viruses during replication [19]. An approach using carbohydrate-binding lectins can be extended to many of the enveloped viruses, given the high degree of similarity between them in terms of the presence of high mannose glycans in envelope glycoproteins. For example, Keyaerts et al. examined a collection of plant ground lectins containing mannose, N-acetyl glucosamine, glucose, galactose, and N-acetyl-galactosamine and found that 15 lectins exerted an anti-SARS-CoV effect [20].

Recently, there has been increased interest in lectins from various marine species, such as algae, sponges, molluscs, fish and arthropods, owing to their value in various medical applications [7]. Algae lectins have unique carbohydrate specificity and physicochemical characteristics compared to other plant lectins and can inhibit the replication of many classes of viruses, including Ebola, influenza A and B, hepatitis C, measles, and herpes simplex type 1 [8]. These include, in particular, lectins isolated from blue-green algae *Cyanobacteria*, such as cyanovirin-N, microvirin, microcystisiride lectin, scitovirin, and lectins from red marine algae [8].

Griffithsin, a lectin from red algae (*Griffithsia* sp.), has a pronounced antiviral activity [21], acting as a potent inhibitor of SARS-CoV infection (strains Urbani, Tor-II, CuHK, and Frank, and is also active against other coronaviruses that infect humans (HCoV (NL63), mammals and birds (infectious bronchitis virus (IBV) [22]. The anti-coronavirus activity of griffithsin is due to its ability to bind both to proteins that are used by viruses as a cellular receptor (for example, SARS-CoV and HCoV-NL63 using ACE2) and carbohydrates (for example, IBV-CoV and HCoV-OC43 using α-2,3-linked sialic acid fragments) [22]. The antiviral potency of griffithsin is likely due to the presence of multiple similar sugar binding sites that provide redundant attachment points for complex carbohydrate molecules present on viral envelopes [13]. It is interesting that these viruses belong to the group 1 and group 3 phylogenetic groups of the *Coronaviridae*, respectively. These data indicate that, at the receptor level, these two viruses have nothing to do except for their susceptibility to inhibition by carbohydrate binding agents [22]. Thus, a wide range of *Coronaviridae* sp. sensitive to griffithsin is an essential attribute of this antiviral protein and determines the prospects for its use in anti-coronavirus strategies. Until now, the effect of griffithsin against coronaviruses has only been tested *in vitro* or in animal models [21],[22]. The next stage in the development of a new drug should be the study of its action in clinical trials.

Overall, glycan-binding lectins are promising antiviral agents, so more research is needed to use them to prevent and control coronavirus infections.

##### 3.1.1.2. Glycosaminoglycan mimetics

Glycosaminoglycans (GAGs), linear sulphated polysaccharides that are present ubiquitously on the cell surface and in the extracellular matrix, are exploited by numerous, distinct microorganisms for cellular attachment, adhesion, invasion and evasion of the host's immune system [23]. SARS-CoV and other coronaviruses also can bind to host cells by clinging through their GAG [24]. GAG mimetics, heparinoid polysaccharides, can also interact with cell surface glycoproteins, causing a shielding effect in these areas and preventing the binding of viruses. Kim et al. (2020) showed recently that heparan sulphate interacts with the GAG-binding motif at the S1/S2 site on each monomer interface in the trimeric SARS-CoV-2 spike glycoprotein and at another site (453–459 YRLFRKS) when the receptor-binding domain is in an open conformation.

The marine environment is a rich source of structurally unique GAGs and GAG-like sulphated glycans [23]. Sulphated fucans of brown algae (*Phaeophyta*) (fucoidan, sargassan, ascofillan and glucuronoxylofucan), sulphated galactans of red algae (*Rhodophyta*) (agar and carrageenan) and sulphated heteropolysaccharides of ulvan containing compounds are examples of these mimetics [25]. Sulphated seaweed polysaccharides show high antiviral activity against enveloped viruses, including important human pathogens such as human immunodeficiency virus, herpes simplex virus, human cytomegalovirus, dengue virus and respiratory syncytial virus [25]. Kwon et al. (2020), using Vero-CCL81 cells that express both ACE2 and TMPRSS24, established the ability of high molecular weight branched sulphated polysaccharides RPI-27 and RPI-28 (fucoidans), extracted from the marine alga *Saccharina japonica*, to strongly bind to the S-protein SARS-CoV-2 *in vitro*. None of the polysaccharides showed toxicity even at the highest concentrations. The most pronounced effect was recorded for the polysaccharide RPI-27, whose EC_50_ was significantly higher than the EC_50_ of remdesivir, which is currently approved for emergency use in severe COVID-19 infections [26]. The authors believe that the higher affinity of RPI-27 compared to RPI-28, and hence its more potent antiviral activity, may be due to the far higher molecular weight of the former, providing greater opportunity for multipoint binding to the S-protein of SARS-CoV-2. The advantage of these polysaccharides is that they can be used via a nasal spray, metered dose inhaler, or oral delivery compared, for example, with remdesivir, which must be administered intravenously or heparin that is not bioavailable orally [26].

Oral intake of fucoidans isolated from seaweed is considered ‘Generally Recognized as Safe’ [26], and drinks, dietary supplements and functional foods based on it are registered in many countries.

Morokutti-Kurz et al. showed the ability of iota-carrageenan to neutralize particles of the pseudotypic lentivirus SARS-CoV-2 spike [27].

Summarizing the above, it can be assumed that, theoretically, a polysaccharide of any alga that mimics GAGs can induce the formation of formally similar complexes that will block the interaction of viruses with cells and weaken cellular infection [23],[26]. The study of marine GAGs ​​mimetics in this direction (in *in vitro* experiments and in clinical trials) will help the development of carbohydrate therapy for coronavirus infections.

#### 3.1.2. Inhibitors of viral lipid-dependent attachment to host cells

Lipids play a central role in viral infection, as they form the structural basis of cell and viral membranes and they are involved in key events in the life cycle of the virus and can act as direct receptors or cofactors of virus entry on the cell surface and in endosomes [28]. Cellular lipid membranes are an important initial point of interaction for viruses that can use microdomains of cell membranes called lipid rafts (membrane rafts) for some stages of their replication cycle. The involvement of membrane rafts in mediating this process has been highlighted for several viruses [29]. A summary of examples of viral-lipid interactions is presented in the review by Chan et al. (2010). To date, the results of several studies also presume the participation of lipid rafts for SARS-CoV, especially during the early replication stage [30]. A recent review by Italian researchers also highlights the role of membrane lipids in the mechanism of SARS-COV-2 infectivity, based on past research on virus attachment to host cells [31]. Detection of virus-lipid interactions expands antiviral therapeutic approaches and may include drugs targeting lipid metabolism.

##### 3.1.2.1. Sterols

Molecules that affect lipids can be used to selectively suppress virus replication. Naturally occurring substances, such as cyclodextrin and sterols, or sphingolipids [32] can reduce the infectivity of many types of viruses, including the coronavirus family, by interfering with lipid-dependent attachment to human host cells [32]. Cyclodextrins, cyclic oligosaccharides, consisting of a macrocyclic ring of glucose subunits joined by α-1,4 glycosidic bonds disrupt the lipid composition of the cell membrane of the host, reducing the attachment of the virus to protein receptors, while phytosterols are mimics of cholesterol that can bind to the virus instead of membrane rafts [33].

Sterols with useful biological activities, including antivirus, have been found in various groups of marine organisms, such as algae, porifera, coelenterata, bryozoa, molluska, echinodermata, arthropoda, tunicata and chordata [34]. A preeminent position is occupied by porifera (i.e., sponges) [34]. For example, Gauvin isolated 5α,8α-epidioxy sterols from the marine sponge *Luffariella variabilis*, which showed inhibitory activity against the HTLV-1 [35]. McKee-evaluated a total of 22 sulphated sterols isolated from marine sponges for their antiviral activity against HIV-1 and HIV-2 [36]. Sterols with sulphate groups at positions 2, 3 or 6 were the most active.

Given these data, it seems necessary to conduct additional broader research of these molecules, as they can become the basis for new anti-coronavirus strategies.

### 3.2. Binding to specific receptors and fusion of cytoplasmic and viral membranes

Proteolytic cleavage of the outer structures of the virus, ensuring the fusion of the membranes of the virus and the cell, is required for the successful attachment of the virus and subsequent penetration into the cell after binding to the receptors [16]. Inhibition of the entry of coronavirus into the cell can be carried out both by substances that specifically interact with the S protein and by blocking of cellular factors, mainly various proteases, necessary for this process [37]. Therefore, the surface proteins of host cells, which can act as receptors for interacting with the virus, and host proteases are potential targets for the search and design of antiviral agents

#### 3.2.1. ACE2 inhibitors

The role of ACE2 in interaction with SARS-CoV has been shown by Li et al. (2003) for SARS-CoV and confirmed by recent studies for SARS-CoV-2 demonstrating that the SARS-CoV-2 spike protein directly binds with the host's cell surface ACE2 receptor, facilitating virus entry and replication [38]. It is noteworthy that coronaviruses from different genera, such as HCoV-NL63 (α-CoV) and SARS-CoV (β-CoV), recognize the same region of ACE2 [39]. These data support the critical role of ACE2 as a specific potential therapeutic target for coronavirus infection, including COVID-19.

##### 3.2.1.1. Peptides

Peptides that mimic ACE2 on the surface of human cells are potential therapeutic agents for containing coronavirus and have several advantages over small molecules (increased specificity) and antibodies (small size) [36]. Potent inhibitors of coronavirus infection, which are short peptides, were derived from sequences in the functional domains in the S-loop of the coronavirus [40].

Marine peptides attract the attention of researchers because of their structural diversity, wide spectrum of therapeutic action, low rate of biological deposition in body tissues, and specificity for targets. These peptides have been isolated from different phyla, such as *Porifera, Cnidaria, Nemertina, Crustacea, Mollusca, Echinodermata* and *Craniata* [41]. Even though the number of antimicrobial peptides with antiviral activity is still low, they already show immense potential to become pharmaceutically available antiviral drugs [42]. A large number of them are already in different phases of the clinical and preclinical pipeline, and others are already used in the treatment of infectious diseases [42]. The mechanism of antiviral action of marine peptides includes blocking of virus entry, inhibition of cytopathic effect, viral neutralization, fusion and entry [43]. Recently, the attention of researchers has been drawn to marine microorganisms as a source of peptides. Almost there is no marine microorganism that does not produce natural antibacterial compounds as an essential line of defense to survive [44]. This class of compounds could also form the basis for new anti-coronavirus strategies.

These data allow us to hope that ACE2 inhibitors can be found among antiviral peptides of a marine source, which will be used as alternative therapeutic agents against CoV infection.

#### 3.2.2. TMPRSS2 inhibitors

##### 3.2.2.1. Flavonoids, terpenes and peptides

SARS-CoV-2 uses TMPRSS2 proteases to efficiently activate the S protein to induce fusion of the virus and cell membranes [38]. Therefore, the development of therapeutic agents targeting TMPRSS2 could be a promising countermeasure against current and emerging coronavirus outbreaks.

Among the currently known natural inhibitors of TMPRSS2 are flavonoids, terpenes, peptides, and coumarins [45]. Marine organisms are also potential sources of TMPRSS2 inhibitors [45].

Terpenes and terpenoids (plant hydrocarbons, the skeleton of which contains isoprene units) have the largest number of representatives and the greatest structural diversity in the kingdom of natural compounds [46]. The structural diversity of terpenoids determines a wide range of biological actions, which makes them interesting as potential drugs. Hemiterpenes, monoterpenes, sesquiterpenes, diterpenes, sesterterpenes and triterpenes are isolated among terpenoids depending on the number of isoprene units. Terpenoids are also some of the most commonly found marine natural products today. Sesterterpenoids and triterpenoids are common, especially in sea sponges, and show marked bioactivity. Some terpenoid compounds are in preclinical or clinical development [46].

Coumarinic compounds are a class of lactones structurally constructed by a benzene ring fused to an α-pyrone ring [47]. A lot of coumarin compounds are medicinal candidates for drugs with strong pharmacological activity, low toxicity and side effects, less drug resistance, high bioavailability, broad spectrum, and better curative effects to treat various types of diseases and are being actively studied [47]. There is much evidence showing its inhibitory role against infection of various viruses, including HIV, influenza, enterovirus 71 (EV71) and coxsackievirus A16 (CVA16). Сoumarin inhibits many of the proteins involved in the transcription/translation machinery required in the life cycle of the virus [47].

Rahman et al. (2020) conducted a virtual screening of natural compounds from the Natural Product Activity and Species Source (NPASS) and identified highly active compounds that inhibit TMPRSS2, the priming agent of SARS-CoV-2, including diterpene excavatolide M, isolated from the soft coral *Formosan gorgonian Briareum*; compound NPC163169, from the algae family *Sargassum*; and decalactone dictyospheric acid A, a coumarin class compound isolated from the green alga *Dictyosphaeria versluyii*.

Pseudotheonamides, which are linear pentapeptides embracing the rare piperazinone and piperidinoiminoimidazolone ring systems, isolated from the marine sponge *Theonella swinhoei* showed good inhibitory activity against serine protease [48].

These data demonstrate that marine peptides and proteins, terpenoids and other components of marine origin can claim the role of compounds capable of blocking viral penetration, inhibiting fusion, and neutralizing viral particles.

### 3.3. Virion deproteinization

After adsorption and fusion of the cytoplasmic and viral membranes, the internal structures of the virus enter the cytoplasm of infected cells, where partial deproteinization of the virion and the release of the internal nucleoprotein take place [49]. Deproteinization is carried out by proteases located on the surface of the cell-TMPRSS2 [49], endosomal cysteine proteases (cathepsins) [50], proprotein convertases during virus assembly in producing cells, and extracellular proteases after the virus leaves the cell. Thus, conditions are created for transcription and replication of the viral genome using its polymerase (transcriptase) complex.

Four non-structural proteins of SARS-CoV (chymotrypsin-like сysteine protease (3CLpro), also known as main protease (Mpro) [51]; papain-like protease (PL2pro); helicase; and RNA-dependent RNA polymerase) are key enzymes in the viral life cycle [16]. PL2pro and 3CLpro are synthesized as large precursor proteins, which are cleaved to form mature active proteins; their structures are preserved across the CoV genera and are involved in the degradation of large polyproteins of the coronavirus. The viral protease 3CLpro is considered the most important of the two, as it is responsible for the release of key viral replicative proteins, including viral RNA polymerase and helicase proteins [51].

#### 3.3.1. CLpro inhibitors

**Figure 1.**
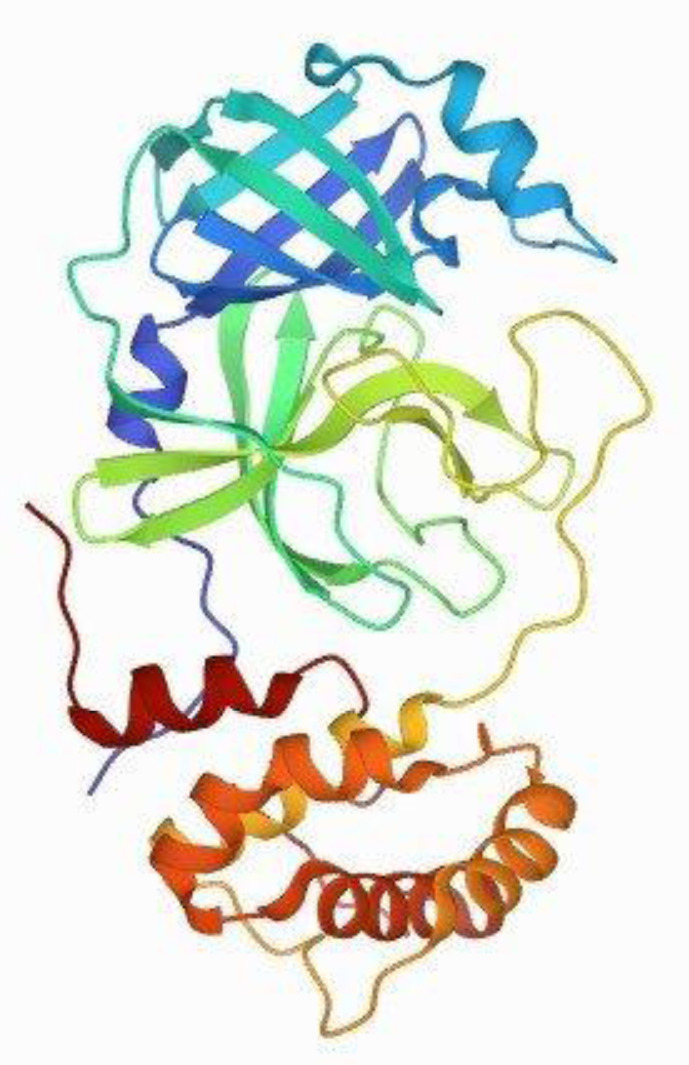
Crystal structure of SARS coronavirus main proteinase (3CLPRO).

The central role of 3CLpro in SARS-CoV replication has made it a prime potential target for antiviral drug development (Figure 1).

Based on the crystal structure of 3CLpro, a variety of inhibitors have been developed in the past 5 years and numerous 3CLpro inhibitors have been reported, including peptide mimetics and small molecules [51],[52]. Lopinavir and ritonavir, which are inhibitors of HIV protease, also inhibit 3CLpro [53]. *In silico* studies directed among commercially available drugs, such as colistin, valrubicin, icatibant, bepotastine, epirubicin, epoprostenol, vapreotide, aprepitant, caspofungin, and perphenazine, also bind to the lopinavir/ritonavir-binding site on CoV [53].

Many research groups have used 3CLpro as a potential drug target for the fight against COVID-19. For example, an international team of researchers has tested more than 10,000 drug molecules already in use in the clinic or in clinical trials and a number of other molecules with pharmacological activity and identified six potential inhibitors of the main protease of the virus that causes COVID-19 [51]. Structural analysis of the enzyme from different coronaviruses showed that the substrate-binding cavities between domains I and II are highly conserved among all 3CLpro, and therefore, an inhibitor that binds to this site will act against all coronaviruses.

Recent reviews [5],[54] summarize information on natural plant-derived 3CLpro inhibitors. Biologically active compounds from marine organisms also demonstrate potential inhibitory activity against RNA viruses [11].

##### 3.3.1.1. Phlorotannins

Polyphenolic compounds of both marine and terrestrial plants are of considerable interest as effective antimicrobial and antiviral agents [55]. Brown algae contain a unique class of polyphenolic compounds called phlorotannins [55]. These compounds are based on the phloroglucinol monomeric unit. Possessing a wide spectrum of biological activities, such as antibacterial, antioxidant, anti-inflammatory, antiproliferative, anticancer, antidiabetic, radioprotective, antiadipogenic, antiviral and antiallergic effects, phlorotannins are considered promising candidates for the development of pharmaceutical agents [55].

Gentile et al. (2020) conducted a virtual screening 164,952 conformers of the 14,064 molecules contained in the library of marine natural products based on consensus high-throughput pharmacophore modelling and molecular docking and identified 17 potential inhibitors of SARS-CoV-2^3CLpro^ among marine natural substances. The results of molecular docking showed that these compounds had docking energies ranging from 4.6 to 10.7 kcal/mol. The most promising inhibitors of SARS-CoV-2^Mpro^ were primarily represented by phlorotannins, which were isolated from the brown alga *Sargassum spinuligerum* [12].

Other types of brown algae also contain large amounts of phlorotannins [56],[57] and may be sources of potential Mpro inhibitors. Park et al. (2013) performed a biological evaluation on nine phlorotannins isolated from the edible brown alga *Ecklonia cava*. The nine isolated phlorotannins (1–9), except phloroglucinol (1), possessed SARS-CoV^3CLpro^ inhibitory activities in a dose-dependent and competitive manner. Of these phlorotannins, two eckol groups with a diphenyl ether linked dieckol showed the most potent SARS-CoV^3CLpro^ trans/cis-cleavage inhibitory effects. Dieckol and 6,6′-bieckol phlorotannins isolated from the edible brown alga *Ecklonia cava* have also been identified as inhibitors of Mpro [12]. It has also been shown that the marine compounds, the structures of which were originally identified by Felix et al. (2017) and related to alkaloids, lipids, terpenoids, and phenols, interacted with the Mpro active site and surrounding residues, forming many hydrogen and hydrophobic interactions [11].

##### 3.3.1.2. Lipids

Мarine organisms (marine bacteria and cyanobacteria, phytoplankton, macroalgae, marine invertebrates, and sponges) produce a variety of lipids. In addition to the major polyunsaturated fatty acids, such as eicosapentaenoic and docosahexaenoic acids, a great number of various fatty acids occur in marine organisms (e.g., saturated, mono- and diunsaturated, branched, halogenated, hydroxylated, methoxylated, and non-methylene-interrupted acids, as well as phospholipids and glycolipids) [60]. The great interest in lipids is associated with their diverse biological activity and direct or indirect participation in many physiological processes. Lipids function as important storage compounds for maintaining cellular activity, affect cell permeability and the activity of many enzymes, participate in intercellular contacts, and in immunochemical processes. In addition, some lipids function as protein modifiers or signalling molecules. Recent studies have established the ability of marine lipids isolated earlier from sea sponges of the *Aplysiidae* family and soft corals (*Pterogorgia citrina*) [59] to affect the main protease of SARS-CoV-2 [11].

##### 3.3.1.3. Terpenoids, lactone

Mechanisms of antiviral action include inhibition of reverse transcriptase and proteases. To date, protease inhibitor drugs, especially HIV-1 protease inhibitors, have been available for human clinical use in the treatment of coronaviruses [61]. In this regard, the search for natural bioactive compounds that were obtained from bioresources that exert inhibitory capabilities against HIV-1 protease activity is of great interest. Among promising protease inhibitors, for example, are new diterpenes isolated from the Brazilian brown alga *Dictyota pfaffii* [62]. Puupehedione, a compound belonging to the terpenes class and isolated from marine sponge *Petrosia strongylophor* and reported earlier in the study [59], also exhibited a good interaction with viral Mpro [11]. De Lira et al. (2007) screened crude extracts and pure compounds isolated from the sea sponge *Axinella cf. corrugata* and found two coumarin derivatives, esculetin-4-carboxylic acid methyl ester and esculetin-4-carboxylic acid ethyl ester, inhibit SARS-CoV^3CLpro^ *in vitro* and SARS-CoV replication in Vero cells.

##### 3.3.1.4. Alkaloids

Alkaloids constitute one of the main classes of secondary metabolites isolated from marine sponges. They exist in derivatives of several heterocyclic rings and exhibit a wide range of biological activities, including antiviral activity [64]. A high ability to bind to SARS-COV-2^Mpro^ has been established for the alkaloid fistularin-3/11-epifistularin-3 (PubChem CID 11170714)^11^, which was isolated earlier from sea sponges of the family *Aplysinidae* [59].

##### 3.3.1.5. Flavonoids

Another class of promising 3CLpro inhibitors has been identified in flavonoids, a subclass of polyphenols derived from secondary plant metabolites [65]. Flavonoids are also inhibitors of other enzymes, in particular, hydrolases, oxidoreductases, DNA synthases, RNA polymerases, phosphatases, protein phosphokinases, and oxygenases. The multiple effects of flavonoids in cells depend on the ability to exert a modulating effect on various components of intracellular signalling cascades, including the cascades of tyrosine kinases, MAP kinases, and protein kinase C [66].

Marine flavonoids have been widely studied in recent decades due to the growing interest in their promising biological/pharmacological activity [67]. Although most of the flavonoids are hydroxylated and methoxylated, some marine flavonoids possess an unusual substitution pattern not commonly found in terrestrial organisms, namely the presence of sulphate, chlorine, and amino groups. Most of the flavonoids have been isolated from seagrasses and halophytes; nevertheless, the isolation of these natural products have also been reported from other marine sources, such as mangroves, algae, molluscs, fungi, corals, and bacteria [67]. Marine flavonoids have demonstrated potential antiviral activity, including those based on the ability to inhibit viral enzymes [68]. Gentile et al. (2020) showed that flavonoids from the brown alga *Sargassum spinuligerum* (apigenin-7-O-neohesperidoside, luteolin-7-rutinoside, and resinoside) bind to SARS-COV-2^Mpro.^

##### 3.3.1.6. Peptides

Pseudoteonamide D and pseudotheonamide C from the marine sponge *Theonella swinhoe* [59] were also found among the compounds with activity against SARS-CoV-2^Mpro^ [11].

Oligopeptides obtained as a result of *in silico* hydrolysis of 20 marine fish proteins by gastrointestinal enzymes bound to the SARS-CoV-2 pine protease can be used as the main compounds for the development of potential COVID-19 inhibitors [69].

#### 3.3.2. Сathepsin inhibitors

Cathepsins, or endosomal cysteine proteases, act in the low pH environment inside the vesicle, and this causes the viral membrane to fuse with the vesicle membrane. Thus, viral proteins and nucleic acids can enter the cell (where viral replication occurs) [70]. Cathepsin L is involved in the cleavage of the S1 subunit of the surface glycoprotein of the coronavirus spike. This cleavage is necessary for the penetration of the coronavirus into the cells of the human host, fusion of the endosomal membranes of the virus and the host cell, and the release of viral RNA for the next round of replication [50]. While the serine protease TMPRSS2 acts locally at the plasma membrane of the host cell and during endocytotic vesicle trafficking [71], cathepsin L continues to degrade the S1 subunit in the acidic endosome and lysosome compartments [72]. The involvement of endosomal protease activity after interactions with SARS-CoV receptors determines the possibility of using inhibitors of these proteases in infection control strategies. In particular, it is proposed to test the combined inhibition of cathepsin L and the serine protease TMPRSS2 as a new treatment for patients with COVID-19 [72]. The proposed dual protease inhibitor therapy could combat SARS-CoV-2 infections not only at the entry point on the plasma membrane of host cells but also in the endosome, which are serial steps in viral pathogenesis, in addition to preserving adaptive immunity [72]. Several potential therapeutic cathepsin L inhibitor candidates and FDA-approved drugs exist to date that may be effective in treating SARS-CoV-2 infections, including antimicrobial, antimalarial, immunomodulatory agents, and others. These already approved drugs may be redeployed to treat SARS-CoV-2 infections [70].

##### 3.3.2.1. Peptides

Biologically active compounds from marine organisms are promising candidates for the development of cathepsin L inhibitors [73]. In particular, the metabolites of marine cyanobacteria show strong inhibitory activity against various classes of proteases [74]. The peptide gallinamide A (C_31_H_53_N_4_O_7_), a potent selective inhibitor of human cathepsin L, has been identified in marine cyanobacterial extracts [74]. Grassistatin A, Grassistatin B, Grassistatin C, a linear decapipeptides also isolated from marine cyanobacteria, selectively inhibited CatD and E and reduced antigen presentation by dendritic cells [75]. Miraziridine A, a tetrapeptide molecule with the alpha-carboxylic aziridine acid moiety found in the blue marine sponge *Theonella aff. Mirabili* [76] and the red marine sponge *Theonella swinho* [77], showed inhibitory activity against trypsin-like serine proteases, papain-like cysteine proteases, and pepsin-like aspartyl proteases. Also from the marine sponge *Theonella mirabilis*, the cathepsin B inhibitor tokaramide A was isolated and its structure was elucidated [78].

##### 3.3.2.2. Other marine-derived cathepsin inhibitors

Placortide E, obtained from the sea sponge *Plakortis halichondroides*, showed activity against cathepsin-like cysteine proteases [79]. Tetronic acid-based antibiotic teromycins extracted from *Streptomyces axinellae* associated with the sponge *Axinellae polypoides* also inhibited cathepsin L [80].

These data indicate that biologically active compounds from marine organisms are promising candidates for the development of inhibitors of coronavirus viral proteases. However, it should be borne in mind that cathepsin L is not specific for SARS-CoV and may interfere with other pathways associated with normal cellular processes. At the same time, research in this direction will contribute to understanding not only the mechanism of penetration of the SARS-CoV virus but also, possibly, other enveloped viruses [72].

### 3.4. Viral replication

#### 3.4.1. RNA-dependent RNA polymerase inhibitors

The viral genetic material delivered to the cell undergoes replication/transcription [16]. Depending on the type of virus, this can occur in the nucleus, cytoplasm, or both compartments. Coronaviruses, like all positive strand RNA viruses, synthesize viral RNA with the formation of subgenomic mRNA [81]. Subgenomic mRNA direct the synthesis of non-structural proteins, including RNA-dependent RNA polymerase (RdRP). RdRP is an essential viral enzyme in the life cycle of all positive strand RNA viruses, including SARS-CoV and SARS-CoV-2, and catalyses the replication and transcription of the RNA genome [82]. Inhibitors of viral RdRP are drugs that can potentially be used to prevent the multiplication of RNA viruses, including coronaviruses (including MERS and SARS) [83]. Nucleoside analogues in the form of adenine or guanine derivatives have been proposed as inhibitors of viral RdRPs [84]. Remdesivir, favipiravir, tenofivir, and ribavirin are also RdRP inhibitors [8].

Singh et al. (2020) performed a comprehensive molecular docking study with a library of 100 natural polyphenols with potential antiviral properties from the PubChem database (https://pubchem.ncbi.nlm.nih.gov) and showed that EGCG, theaflavin-3′-O-gallate, theaflavin-3′-galla, and theaflavin 3,3′-digallate possess a better binding affinity than the control drug remdesivir against SARSCoV-2 RdRp [85].

Biologically active compounds from marine organisms are also being investigated as possible candidates for the role of inhibitors of viral RdRP. For example, Yang et al. screened about 800 molecules from marine microorganisms and crude extracts obtained from aquatic organisms and identified a compound isolated from marine fungi that inhibits the NS3 helicase and NS5B RdRP of the hepatitis C virus.

Although coronaviruses do not have reverse transcriptase, the use of inhibitors of this enzyme in conjunction with other antiviral drugs for the treatment of COVID-19 is being studied [87]. In this regard, attention should be paid to sulphated fucans from the algae, which are capable of inhibiting the activity of HIV reverse transcriptase (RT), herpes virus, respiratory syncytial virus, cytomegalovirus and dengue virus [88].

### 3.5. The exit of virions from the cell

#### 3.5.1. Viral ion channel inhibitors

The mechanisms of final assembly and release of virions may be another possible point of application of target-specific compounds in viral infections. One of the mechanisms is associated with the inhibition of ion channels. Viral ion channels are short membrane proteins with 50–120 amino acids and play an important role either in regulating virus replication, such as virus entry, assembly and release, or modulating the electrochemical balance in the subcellular compartments of host cells [89]. In *Coronaviridae*, it was demonstrated that the envelope (E) proteins of MHV, SARS-CoV, HCoV-229E and IBV exhibit viroporin activity, which modify cell membranes, thereby facilitating the release of the virus from infected cells [90]. The SARS-CoV E protein is more selective for Na+ than for K+ ions [89]. It was also found that another protein, SARS-CoV 3a, can form an ion channel and modulate virus release [91].

In this regard, natural molecules can be potentially used as modulators of ion channel functions. The main group of natural products that dominate the pharmacology of ion channels are peptides/polypeptides. Non-peptide natural products targeting ion channels remain largely unexplored but should become much more affordable in the coming decades [92]. It has been shown, for example, that the flavanols kaempferol, kaempferol glycosides and acylated derivatives of kaempferol glucoside isolated from medicinal herbs inhibit the formation of cation-selective ion channels formed during the reproduction of SARS-СoV involved in the release of virions from the cell [93].

Marine resources represent a huge untapped potential for the discovery of future pharmacologically active marine compounds targeting ion channels. Poisons of marine aquatic organisms have become the sources of such compounds in recent decades. Comprehensive information on the structural and functional properties of marine toxins that interact with ion channels are summarized in the reviews [94],[95]. These compounds are very diverse chemically and include peptides, polypeptides, alkaloids, cyclic polyethers, esters, and heterocycles. Teichert et al. (2010) believe that many of the future pharmacologically active marine compounds targeting ion channels will be non-peptide organic compounds that have evolved to cross membrane barriers in the marine environment. It should be added that ion channels have very important functions in cells. Since inhibitors that block viral ion channels can be potentially harmful to cells, it is better to use them for a short period of time to temporarily reduce the viral titre *in vivo*, but not be considered for long-term treatment [89]. However, possible chemical modifications can reduce the toxicity of these potential drugs, which can be used in anti-coronavirus strategies.

## 4. Conclusions

Today, marine natural products remain as a rich source of novel therapeutic agents for the treatment of different human illnesses [96],[97]. The enormous potential of marine resources and the need to search for new therapeutic agents for the treatment of various diseases, including infectious diseases, are a stimulus for the study of marine compounds in experiments and clinical trials. Viral infections remain a serious threat to human health, and researchers around the world are turning to the ocean for original structures that can form the basis of new antiviral strategies. Most antiviral drugs are designed to block the function of critical viral proteins, which is unique to a particular virus or family of viruses [3]. At the same time, the lack of broad-spectrum antiviral drugs aimed at blocking the targets of host cells and cancelling the replication of many viruses creates a large gap in preparedness for emergency situations with viral infectious diseases. The development of such drugs previously was met with scepticism, mainly due to problems with toxicity and poor translation in an *in vivo* model. The idea of a drug that can effectively treat a wide range of viral infections by blocking specific host functions has emerged with the advent of new and more effective screening methods and predictive tools [4]. The rapid response to new or changing pandemic threats can be aided by the availability of an arsenal of antiviral drugs with overlapping therapeutic options that have the potential for treating emerging agents [98]. Screening for natural and derived bio-active compounds in preclinical and clinical studies is one of the frontlines of fighting the coronaviruses pandemic [96]. In this regard, the high structural similarity between SARS-CoV-2 and SARS-CoV or MERS-CoV and their similar clinical manifestations suggest that these viruses will respond in a similar way to therapeutic drugs.

Numerous compounds of various structural classes, including polysaccharides, terpenes, steroids, alkaloids and peptides that inhibited both RNA and DNA viruses have been isolated from marine organisms. These substances are able to block the penetration of coronavirus into the cell, inhibit the fusion and neutralize viral particles, inhibit viral proteins, disrupt the replication of the viral genome, affect the release of virions from cells and act on the cellular targets of the host. Some chemical classes, such as flavonoids, alkaloids, lectins, terpenes, polysaccharides, GAGs and peptides, have been successfully tested against COVID-19. The diversity of these chemical classes is related to the different mechanism used by each of them to inhibit coronaviruses. Sources of new pharmacological compounds of marine origin are bacteria, algae, invertebrates (sponges, ophiuras, echinoderms, molluscs, soft corals, bryozoans, tunnels and other marine organisms).

Overall, the analysed research results gives hope that biologically active substances of marine origin can form the basis for new anti-coronavirus strategies. These natural compounds can be important complementary drugs in the fight against viruses due to their natural origin, safety and low cost compared to synthetic drugs. A broad initiative to discover and test new biologically active compounds from marine organisms targeting potential antiviral targets should significantly accelerate progress in the fight against coronavirus infections. Combining the efforts of organic chemists, specialists in the field of molecular and cell biology, pharmacologists and other scientists, the use of interactive multi-user computer support systems for organizing networked scientific research in the analysis of molecular structure-biological activity relationships should help solve this problem.
